# Ultra-rapid auxin metabolite profiling for high-throughput mutant screening in Arabidopsis

**DOI:** 10.1093/jxb/ery084

**Published:** 2018-03-03

**Authors:** Aleš Pěnčík, Rubén Casanova-Sáez, Veronika Pilařová, Asta Žukauskaitė, Rui Pinto, José Luis Micol, Karin Ljung, Ondřej Novák

**Affiliations:** 1 Umeå Plant Science Centre, Department of Forest Genetics and Plant Physiology, Swedish University of Agricultural Sciences, Umeå, Sweden; 2 Laboratory of Growth Regulators, Centre of the Region Haná for Biotechnological and Agricultural Research, Faculty of Science of Palacký University & Institute of Experimental Botany of the Czech Academy of Sciences, Šlechtitelů, Olomouc, Czech Republic; 3 Department of Analytical Chemistry, Faculty of Pharmacy in Hradec Králové, Charles University in Prague, Heyrovského, Hradec Králové, Czech Republic; 4 Computational Life Science Cluster (CLiC), Chemistry department (KBC), Umeå University, Umeå, Sweden; 5 Instituto de Bioingeniería, Universidad Miguel Hernández, Campus de Elche, Elche, Alicante, Spain

**Keywords:** *Arabidopsis thaliana*, auxin, metabolite profiling, multivariate data analysis, mutant, screening

## Abstract

Auxin (indole-3-acetic acid, IAA) plays fundamental roles as a signalling molecule during numerous plant growth and development processes. The formation of local auxin gradients and auxin maxima/minima, which is very important for these processes, is regulated by auxin metabolism (biosynthesis, degradation, and conjugation) as well as transport. When studying auxin metabolism pathways it is crucial to combine data obtained from genetic investigations with the identification and quantification of individual metabolites. Thus, to facilitate efforts to elucidate auxin metabolism and its roles in plants, we have developed a high-throughput method for simultaneously quantifying IAA and its key metabolites in minute samples (<10 mg FW) of *Arabidopsis thaliana* tissues by in-tip micro solid-phase extraction and fast LC–tandem MS. As a proof of concept, we applied the method to a collection of Arabidopsis mutant lines and identified lines with altered IAA metabolite profiles using multivariate data analysis. Finally, we explored the correlation between IAA metabolite profiles and IAA-related phenotypes. The developed rapid analysis of large numbers of samples (>100 samples d^–1^) is a valuable tool to screen for novel regulators of auxin metabolism and homeostasis among large collections of genotypes.

## Teaser

A high-throughput screening of auxin metabolites in combination with multivariate data analysis can be used to identify novel regulators of the IAA metabolism in the model plant *Arabidopsis thaliana*.

## Introduction

**Fig. 1.**
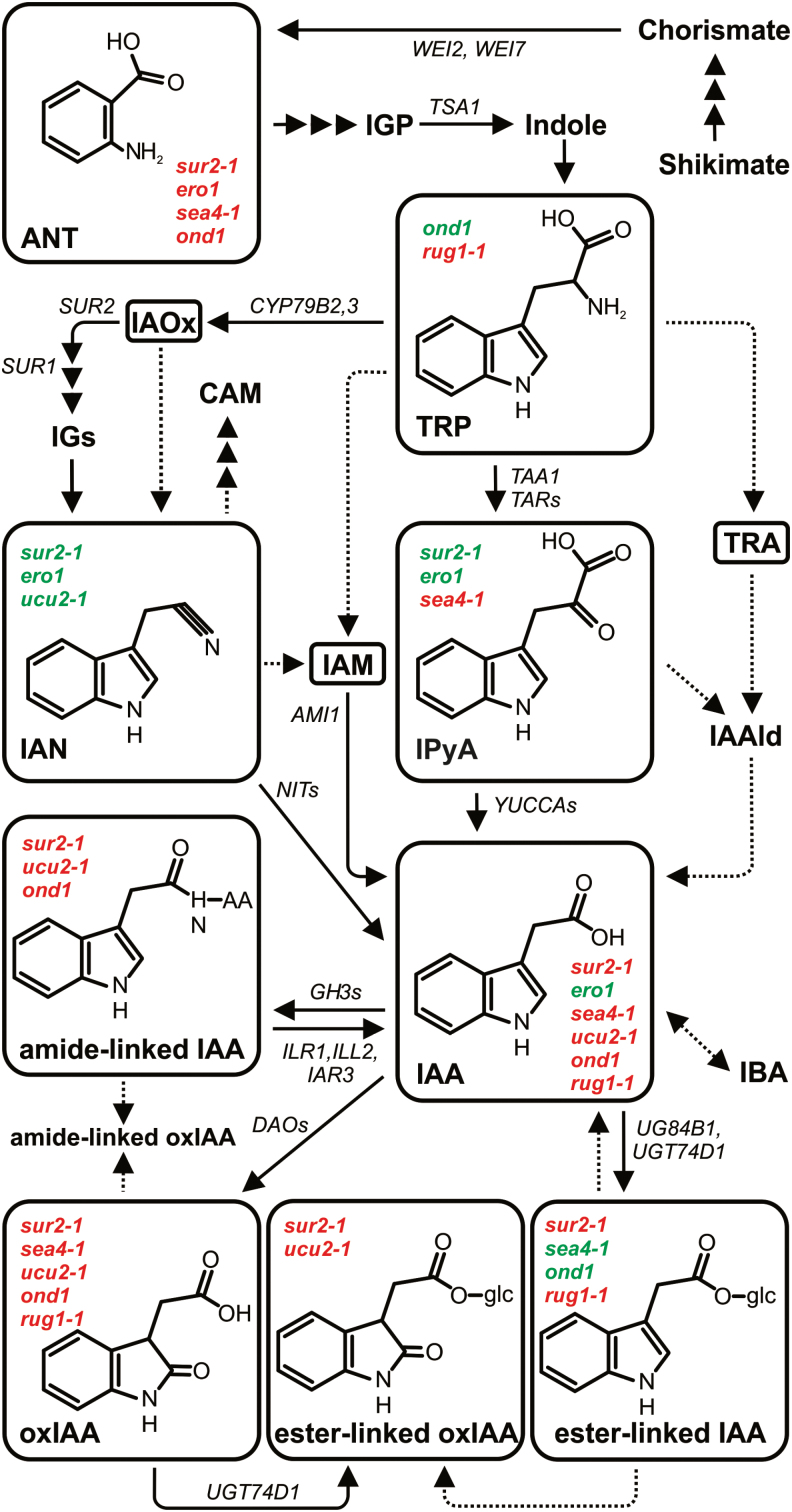
Putative pathways of IAA biosynthesis and metabolism in Arabidopsis. Pathways are based on Ljung (2013), Mano and Nemoto (2012), Kasahara (2016), Ludwig-Müller (2011), and Porco *et al*, 2016. Dashed arrows indicate steps in which the enzymes catalysing the reaction are not known. Significantly higher (red) or lower (green) concentrations of individual metabolites in investigated mutant lines showing the greatest difference from wild-type IAA metabolomes are indicated.

Auxin (indole-3-acetic acid, IAA) plays major roles as a signalling molecule in numerous plant growth and development processes. A crucial step in many of these processes is the formation of local auxin gradients and maxima/minima within plant tissues (Benková *et al.*, 2003), through tightly regulated interplay between biosynthesis, conjugation, degradation, and directional transport of auxin (Rosquete *et al.*, 2012).

l-Tryptophan (Trp), an amino acid generated by the shikimate pathway, is the key precursor in four major auxin biosynthesis pathways in plants: the indole-3-acetamide (IAM), indole-3-acetaldoxime (IAOx), tryptamine (TRA), and indole-3-pyruvic acid (IPyA) pathways, named according to the major intermediate (Fig. 1; for reviews, see Mano and Nemoto, 2012; Ljung, 2013; Kasahara, 2016). These pathways are believed to be the main sources of *de novo* synthesized auxin, but Trp-independent pathways may also exist (Tivendale *et al.*, 2014; Wang *et al.*, 2015). Following biosynthesis, and often following transport, auxin may be degraded by oxidation and subsequent conjugation, yielding the major metabolites 2-oxindole-3-acetic acid (oxIAA) and oxIAA-glucose (oxIAA-glc) (Östin *et al.*, 1998; Kai *et al.*, 2007*a*; Pěnčík *et al.*, 2013).

Another auxin inactivation mechanism is the formation of conjugates with amino acids or sugars (Tam *et al.*, 2000; Kowalczyk and Sandberg, 2001). Some of these conjugates might be hydrolysed, releasing free active auxin, indicating that they may serve as temporary storage forms of the inactive hormone (reviewed by Ludwig-Müller, 2011). However, in *Arabidopsis thaliana*, the most abundant amide-linked conjugates, IAA-aspartate (IAAsp) and IAA-glutamate (IAGlu), are not reversibly converted to IAA, and probably serve as degradation intermediates (Kowalczyk and Sandberg, 2001; Woodward and Bartel, 2005). Recent research suggests that conjugation of IAA to amino acids could play important roles in specific developmental processes (Zheng *et al.*, 2016; Di Mambro *et al.*, 2017). Nevertheless, the roles and regulation of the different pathways of auxin biosynthesis and degradation/conjugation are not well understood.

To improve understanding of auxin metabolism in specific tissues or processes, information on levels of the free hormone, its biosynthetic precursors, and its major metabolites is highly important. However, analysing plant hormones is challenging due to their very low concentrations and the complexity of plant extracts (Tarkowská *et al.*, 2014). Furthermore, many of these compounds are unstable and can be easily degraded during extraction and purification. Fortunately, some of the problems associated with phytohormone analysis can be overcome by exploiting recent advances in analytical techniques, such as ultra-fast LC coupled with high-sensitivity tandem MS (LC-MS/MS) (Novák *et al.*, 2014; Porfírio *et al.*, 2016). For samples containing minute amounts of tissue, the sensitivity of the analytical method can also be improved by miniaturization of the extraction and purification steps, which can minimize analyte losses due to adsorption to surfaces and/or increase analyte recovery in the solid phase extraction (SPE) step (Svačinová *et al.*, 2012; Novák *et al.*, 2017). Micro-purification techniques have previously been used for auxin isolation, and these include solid phase microextraction (SPME) (Liu *et al.*, 2007), in-tip micro-SPE (µSPE) (Liu *et al.*, 2012), and magnetic SPE (Zhang *et al.*, 2010). Nevertheless, further advances are still required.

There is a particular need for a simple, high-throughput analytical approach that provides sufficient robustness, sensitivity, and selectivity for analyses of large numbers of samples, such as from mutant collections. In the study presented here, an ultra-rapid auxin profiling method, involving micro-scale in-tip SPE (in-tip µSPE) and ultra-fast LC-MS/MS analysis, was developed and evaluated. The high-throughput approach was successfully validated against the previously published purification protocol using a polymer-based reversed phase sorbent (Novák *et al.*, 2012). The method was then used for screening a collection of Arabidopsis mutant lines (Berná *et al.*, 1999; Pérez-Pérez *et al.*, 2009), and several lines with altered IAA metabolite profiles were identified using multivariate data analysis (MVDA). Compared with previously published methods, the approach presented here is less time-consuming and allows greater processivity (>100 samples d^–1^). This makes it an ideal tool for the screening of large collections of lines, and subsequent identification of altered metabolite profiles, which will ultimately help researchers to find novel players in specific IAA metabolic pathways.

## Materials and methods

### Reagents and standards

Standards for IAA and its metabolites anthranilate (ANT), indole-3-acetaldehyde (IAAld), IAM, indole-3-acetonitrile (IAN), IPyA, TRA, and Trp were purchased from Sigma-Aldrich (http://www.sigmaaldrich.com), and standards for IAOx and oxIAA from Olchemim Ltd. (http://www.olchemim.cz/). Unlabelled IAAsp and IAGlu, their indole-^13^C_6_-labelled forms, and 2-oxo-[indole-^13^C_6_]IAA were synthesized as described by Ilić *et al.* (1997) and van de Weert *et al.* (1998), with the modifications described by Kowalczyk and Sandberg (2001). Unlabelled and indole-^13^C_6_-labelled IAA-glc and oxIAA-glc were synthesized using modifications of literature procedures previously described by Kai *et al.* (2007*b*). [Benzyl-^13^C_6_]ANT and [indole-^13^C_6_]IAA were obtained from Cambridge Isotope Laboratories (http://www.isotope.com), and [β, β-^2^H_2_]TRA and [indole-^2^H_5_]Trp from C/D/N Isotopes (https://www.cdnisotopes.com). Labelled IAAld, IAM, and IAN were synthesized from the methyl ester of unlabelled IAA or [^13^C_6_]IAA using the method described by Kowalczyk (2002). [Indole-^2^H_5_]IAOx and [indole-^2^H_4_]IPyA were synthesized as described by Novák *et al.* (2012). Acetic acid was purchased from Merck (http://www.merck.com); diethyldithiocarbamic acid sodium salt and cysteamine hydrochloride from Sigma-Aldrich; Murashige and Skoog medium from Duchefa (http://www.duchefa.com), and HPLC gradient grade solvents from J.T. Baker – Fisher Scientific (https://www.fishersci.com). All other chemicals were from Lach-Ner (http://www.lach-ner.com) and Sigma-Aldrich.

### Plant material and growth conditions

Seven-day-old Arabidopsis wild-type seedlings were used as the material for development and validation of the ultra-rapid auxin metabolite profiling method. The 64 Arabidopsis leaf mutants used in this study were isolated in the laboratory of José Luis Micol and have been described by Berná *et al.* (1999) and Pérez-Pérez *et al.* (2009). The *sur2-1* mutant line (Barlier *et al.*, 2000) was included as a positive control. *Arabidopsis thaliana* Columbia-0 (Col-0) and Landsberg *erecta* (L*er*) wild-type accessions were obtained from the Nottingham Arabidopsis Stock Centre (NASC). All seeds were surface-sterilized using a bleach solution containing 0.002% Triton X-100 and then sown on Murashige and Skoog square agar plates (4.4 g l^–1^ Murashige and Skoog, 0.5 g l^–1^ MES monohydrate, and 8 g l^–1^ plant agar, pH 5.7). After 3 d of stratification, the plates were placed vertically in long-day conditions (16 h light/8 h dark) at 22 ± 1°C under cool white fluorescent light (maximum irradiance 550 μmol m^−2^ s^−1^). Whole seedlings were collected in five replicates and weighed, immediately frozen in liquid nitrogen, and stored at –80 °C until extraction.

### Extraction and purification of IAA metabolites

Frozen samples were placed in a crushed-ice bath in order to avoid enzymatic degradation of analytes. For quantification of IAA and its metabolites, samples containing 10 mg (FW) of plant material were extracted in 1 ml of ice-cold Na-phosphate buffer (50 mM, pH 7.0, 4 °C) containing 0.1% diethyldithiocarbamic acid sodium salt. The following stable isotope-labelled internal standards were added to each sample: [^13^C_6_]IAAsp, [^13^C_6_]IAGlu, [^13^C_6_]ANT, [^13^C_6_]IAA, [^13^C_6_]IAM, [^2^H_5_]IAOx, [^2^H_2_]TRA, [^13^C_6_]oxIAA, [^13^C_6_]IAA-glc, and [^13^C_6_]oxIAA-glc (all at 2.5 pmol per sample); [^2^H_4_]IPyA and [^13^C_6_]IAN (5 pmol per sample); and [^2^H_5_]Trp (50 pmol per sample). The samples were homogenized using a MixerMill MM 301 bead mill (Retsch GmbH; http://www.retsch.com) at a frequency of 29 Hz for 6 min after adding 2 mm ceria-stabilized zirconium oxide beads. The plant extracts were incubated at 4 °C with continuous shaking (10 min), centrifuged (15 min, 23000 *g* at 4 °C), and purified by in-tip µSPE using self-packed multi-StageTip columns prepared according to Svačinová *et al.* (2012). The columns contained two types of extraction sorbents (three layers of each type): C_18_ and SDB-XC (Empore™, 3M™; http://www.3m.com).

A volume of 200 μl of each plant extract was acidified to pH 2.7 with 0.1 M hydrochloric acid (~100 μl) and loaded onto a multi-StageTip column that had been activated with 50 μl of acetone (by centrifugation at 2200 rpm, 10 min, 4 °C), 50 μl of methanol (2200 rpm, 10 min, 4 °C), and 50 μl of water (2200 rpm, 15 min, 4 °C). After sample application (3400 rpm, 25 min, 4 °C), the column was washed with 50 μl of 0.1% acetic acid (3400 rpm, 15 min, 4 °C) then eluted with 50 μl of 80% methanol (3400 rpm, 15 min, 4 °C). Another 200 μl of the extract was derivatized by adding 100 μl of 0.75 M cysteamine (pH 8.2) to convert the labile compounds IAAld and IPyA to their respective thiazolidine derivatives IAAld-TAZ and IPyA-TAZ (Novák *et al.*, 2012). After 15 min incubation, the sample was adjusted to pH 2.7 and purified as described above. Both eluates were pooled into one vial, evaporated to dryness *in vacuo*, and stored at –20 °C until LC-MS/MS analysis.

Multi-StageTips with C_18_/C_8_ and C_18_/SDB-RPS combinations of sorbent types were also prepared for development of the purification method. Briefly, microcolumns of both kinds were activated sequentially with 50 μl each of acetone, methanol, and water (by centrifugation at 2200 rpm, 10–15 min, 4 °C), and aliquots of the acidified sample extract were applied (3400 rpm, 25 min, 4 °C). The microcolumns were then washed with 50 μl of 0.1% acetic acid (3400 rpm, 15 min, 4 °C), and samples were eluted from the C_18_/C_8_ and C_18_/SDB-RPS sorbents with 50 μl of 80% methanol and 50 μl of 0.5 M NH_4_OH in 80% (v/v) methanol (3400 rpm, 15 min, 4 °C), respectively. To validate the final μSPE protocol, extracts were also purified on Oasis HLB columns (30 mg, Waters Corp., Milford, MA, USA), conditioned with 1 ml of methanol, 1 ml of water, and 0.5 ml of Na-phosphate buffer (pH 2.7) as described by Novák *et al.* (2012). After sample application, the columns were washed with 2 ml of 5% methanol and then eluted with 2 ml of 80% methanol. All eluates were evaporated to dryness and stored as described above.

### Quantification of IAA metabolites

The evaporated samples were dissolved in 40 μl of mobile phase prior to LC-MS/MS analysis, using a 1290 Infinity LC system and a 6490 Triple Quadrupole LC/MS system equipped with Jet Stream and Dual Ion Funnel systems (Agilent Technologies, http://www.home.agilent.com). A 20 μl portion of each sample was injected onto a reversed-phase column (Kinetex C18 100A, length 50 mm, diameter 2.1 mm, particle size 1.7 μm; Phenomenex, http://www.phenomenex.com), and the analytes were eluted by a 3 min linear gradient of 5:95 to 35:65 A:B, where A and B are 0.1% acetic acid in methanol and 0.1% acetic acid in water, respectively. The column was then washed with 100% methanol (1.0 min), and re-equilibrated to initial conditions (1.0 min). Throughout the procedure, the flow rate was 0.5 ml min^−1^, and the column temperature 40 °C. The effluent was introduced into the MS system with the optimized settings listed in Supplementary Table S2 at *JXB* online. Analytes were quantified using diagnostic multiple reaction monitoring (MRM) transitions of precursor and appropriate product ions using optimal collision energies and 50 ms dwell time (Table S2). Chromatograms were analysed using MassHunter software (version B.05.02; Agilent Technologies), and the compounds were also quantified by standard isotope dilution analysis (Rittenberg and Foster, 1940).

### Experimental design and data analysis

Seeds from all Arabidopsis mutant and L*er* wild-type lines were sown in five rows per plate, with 50–70 seeds per row. Plates containing the L*er* seedlings were randomly placed along the racks, so that any growth variation due to the position of the plate on the growth room shelves would be represented in the reference data. Replicates containing ~10 mg (FW) of 7-day-old seedlings were harvested. For each mutant line, including the *sur2-1* positive control, five biological replicates were harvested, while the L*er* wild type was represented by 40 replicates.

Multivariate data analysis was conducted using ‘Soft Independent Modeling of Class Analogies’ (SIMCA) software version 13 (Umetrics AB, Umeå, Sweden). Clustergrams were drawn in R software.

### Plant phenotyping

For plant phenotyping, plates containing vertically grown 10-day-old seedlings were scanned. The digital images were used for primary root and hypocotyl length measurements using FIJI software. The number of lateral roots was counted from the images.

## Results and Discussion

### Development of a µSPE purification method for IAA metabolites

One of the most crucial steps in the development and optimization of a purification method for metabolite profiling is selection of suitable SPE sorbents. Ideally, they should afford good retention and high recovery of all target compounds (which may have diverse chemical properties), while excluding others. They must also be compatible with any miniaturized equipment to be used. Here we made our own stop-and-go extraction tips (StageTips) from ordinary pipette tips containing very small disks made of beads with reversed-phase, cation-exchange, or anion-exchange surfaces immobilized on a Teflon mesh (Rappsilber *et al.*, 2003). We previously showed that auxin metabolites can be efficiently retained by reversed-phase sorbents (Novák *et al.*, 2012). Therefore, two sorbents with long alkyl chains (octyl, C_8_; and octadecyl, C_18_) and two poly(styrenedivinylbenzene) co-polymer-based sorbents (SDB-RPS and SDB-XC) were selected and used for testing in the study presented here.

**Fig. 2.**
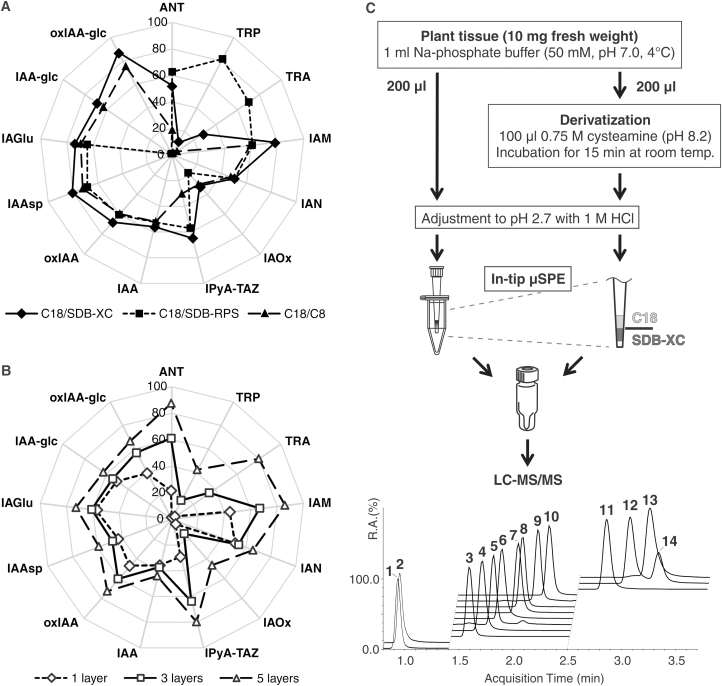
Ultra-rapid auxin metabolite profiling method. (A) Recoveries (%) of IAA metabolites applied to multi-StageTips using Empore sorbents in the indicated combinations (C18/C8, C18/SDB-RPS, and C18/SDB-XC). (B) Recoveries (%) of the indicated IAA metabolites with one, three, and five sorbent multilayers (C18/SDB-XC) used in the µSPE purification procedure. Values are means ±SD (*n*=4). (C) Plant material (10 mg) was homogenized and extracted in Na-phosphate buffer containing labelled internal standards. One portion (2 mg FW per 200 μl) of acidified supernatant was directly applied to a pre-conditioned multi-StageTip microcolumn (STop And Go Extraction Tip created by packing C18/SDB-XC sorbents in an ordinary pipette tip and inserting into a 1.5 ml microcentrifuge tube, which was then washed and eluted with methanolic solutions). The other half of the supernatant (200 μl) was derivatized using cysteamine and also purified by in-tip μSPE. The pooled eluate was evaporated to dryness, dissolved in 40 μl of 5% acidified methanol, and finally analysed using the LC-MS/MS method presented herein, affording ultra-fast chromatographic separation of 14 IAA precursors, catabolites, and conjugates (1, Trp; 2, TRA; 3, IPyA-TAZ; 4, ANT; 5, oxIAA-glc; 6, IAM; 7, IAAsp; 8, oxIAA; 9, IAA-glc; 10, IAGlu; 11, IAA; 12, *trans*-IAOx; 13, IAN; 14, *cis*-IAOx).

Accordingly, IAA and its precursors and degradation products were retained and eluted with varying efficiency (Fig. 2A). A combination of C_18_/C_8_ sorbents retained 19–75% of all of the IAA metabolites, except the most polar compounds (TRA and Trp). StageTips combining C_18_ and SDB-based sorbents provided the highest extraction yields. Because SDB-RPS modified with sulphonic acid groups allows reversed-phase and cation-exchange interactions, amine-containing basic analytes, ANT, TRA, and Trp, were enriched using C_18_/SDB-RPS columns with high recoveries: 63 ± 1, 70 ± 2, 82 ± 1, and respectively. However, recoveries of IAA-glc and oxIAA-glc were far too low with these columns (1.6 ± 0.1% and 1.0 ± 0.1%, respectively), due to degradation of these conjugates under the alkaline conditions (Supplementary Table S1) required for elution from the SDB-RPS sorbent. In contrast, use of C_18_/SDB-XC columns, which do not exploit the cation-exchange interactions, allowed maximization of yields from the in-tip µSPE step and minimization of losses due to pH lability (Fig. 2A). Moreover, the C_18_/SDB-XC combination retained the polar compounds (TRA and Trp) more strongly than the reversed-phase sorbents, C_18_/C_8_; this combination allows lower recovery of Trp during the purification step (10 ± 2%), but this is not limiting due to the very high endogenous levels of Trp in plants (Novák *et al.*, 2012). Therefore, we decided to optimize the one-step purification protocol for multi-µSPE columns further by using a combination of C_18_ and SDB-XC sorbents.

In order to minimize contamination with non-polar extractable substances that could interfere with subsequent MS-based analyses (e.g. pigments and lipids), we used 50 mM Na-phosphate buffer (pH 7.0) as the extraction solvent (Edlund *et al.*, 1995). To maximize recoveries of the analytes when using the µSPE-based approach, we also optimized the loading conditions, by assessing the effects of variations in conditions on analytes classified in terms of their stability and acidity or basicity (in three classes: basic, neutral, and acidic). As shown in Supplementary Fig. S1, we obtained higher recovery under acidic conditions (pH 2.7) for most metabolites (mean recovery, 75 ± 25%, with overall means of 75, 65, and 90% for the basic, neutral, and acidic compounds, respectively) than under neutral pH. As expected, yields of the neutral IAA precursor IAOx were higher in the extraction buffer with neutral pH (96 ± 3%) than under acidic conditions (31 ± 1%), in accordance with the previously reported lability of IAOx in strongly acidic solutions (Novák *et al.*, 2012).

IPyA has also previously exhibited instability in solution, therefore derivatization is required for its accurate quantification (Tam and Normanly, 1998; Mashiguchi *et al.*, 2011). For this purpose, we converted IPyA to the thiazolidine product IPyA-TAZ by derivatization with cysteamine following Novák *et al.* (2012), but miniaturized the derivatization step by using 200 μl of crude plant extract (from ~2 mg FW of plant tissue) with 100 μl of 0.75 M cysteamine (pH 8.2). This afforded 15-fold greater yields of IPyA-TAZ from minute samples than the original derivatization protocol (Novák *et al.*, 2012).

We then tested the influence of a complex multicomponent plant matrix on recoveries of diverse IAA metabolites in small amounts of plant tissue. The efficiency of the entire developed method was evaluated by spiking 10-day-old Arabidopsis seedling extracts with a mixture of stable isotope-labelled standards. We first examined the extraction capability of multi-StageTip columns packed with one, three, and five layers of each sorbent type (C_18_ and SDB-XC) using a crude extract from 2.5 mg FW of plant tissue. As shown in Fig. 2B, recoveries increased as the number of sorbent multilayers increased (mean total extraction yields were ~30, 40, and 60% with one, three, and five bi-layers, respectively). Thus, IAA metabolites were most effectively enriched by using microcolumns packed with five layers of each sorbent (C_18_ and SDB-XC). However, these columns were most prone to clogging due to the high number of layers (10 in total), which hindered the subsequent washing (0.1% acetic acid) and elution (80% methanol) steps. Consequently, multi-StageTips packed with three layers were used in further analyses, and their capacity to isolate IAA metabolites was tested by using them to purify extracts from four different amounts of fresh Arabidopsis tissue (1.0, 2.5, 5.0, and 7.5 mg). As expected, recoveries declined with increasing amounts of fresh plant tissue from overall means of 61 ± 22% for extracts from 1.0 mg to 26 ± 11% for extracts from 7.5 mg, due to overloading of the sorbents (Supplementary Fig. S2A). We concluded that 200 µl of an Na-phosphate buffer extract containing 2 mg FW of plant material was optimal for purifying IAA and its key metabolites using a C_18_/SDB-XC multi-StageTips microcolumn. More than 100 samples can be extracted and purified per working day using this approach. The final method for high-throughput extraction and purification of Arabidopsis samples is shown in Fig. 2C.

### A rapid LC-MS/MS method for IAA metabolite profiling

The most suitable and widely used analytical techniques for auxin analysis currently available are based on MS (Matsuda *et al.*, 2005; Kai *et al.*, 2007*a*; Pěnčík et al., 2009; Sugawara *et al.*, 2009; Mashiguchi *et al.*, 2011; Floková *et al.*, 2014). Recent increases in their sensitivity and selectivity enable tissue- and cell-specific quantification of IAA and diverse IAA metabolites (Novák *et al.*, 2012; Pěnčík *et al.*, 2013). However, in order to process hundreds of samples from Arabidopsis mutant screens, a high-throughput method for auxin profiling was needed. Thus, we combined the micro-scale purification method with rapid, highly sensitive, and selective quantification, using LC-MS/MS. Using a Kinetex™ column with core-shell technology, IAA and 14 precursors, catabolites, and conjugates were separated under optimized conditions (listed in Supplementary Table S2) in just 3.5 min (Fig. 2C). Under these conditions, retention time stability ranged between 0.07% and 0.86% RSD (relative standard deviation), and chromatographic runs were split into three targeted scan windows (0.8–1.4, 1.4–2.5, and 2.5–3.7 min).

Most of the precursor and product ions of IAA metabolites determined under optimized LC-MS/MS conditions corresponded well with previously published data (Novák *et al.*, 2012). Moreover, unlabelled and labelled IAA-glc and oxIAA-glc were included in the IAA profiling, and detected in negative-ion as well as positive-ion MRM mode. In accordance with previously published MS/MS patterns (Kai *et al.*, 2007*a*), IAA and oxIAA ions (*m/z* 174 and 190, respectively) were detected as high-intensity fragments of IAA-glc and oxIAA-glc in negative-ion mode. However, it is well known that negative ion mode (ESI–)-MS is generally less sensitive than positive ion mode (ESI+) (see Supplementary Fig. S3). In efforts to increase sensitivity, we also examined ionization patterns of IAA-glc conjugates in (ESI+)-MRM mode. The neutral losses of a sugar moiety (162 Da) through in-source fragmentation of precursor ions (*m/z* 338 of IAA-glc and *m/z* 354 of oxIAA-glc) produced high-intensity ions, and subsequent fragmentations in the quadrupole collision cell led to the creation of quinolinium/quinolonium ions (*m/z* 130 and 146, respectively) (Kowalczyk and Sandberg, 2001). Thus, MRM transitions *m/z* 176 >130 and *m/z* 192 >146 were used to detect IAA/IAA-glc and oxIAA/oxIAA-glc, respectively. Moreover, both molecule pairs were fully resolved under our reversed-phase LC conditions (Fig. 2C; Supplementary Table S2).

To determine the limits of detection (LODs) and linear calibration ranges of the ultra-fast LC-MS/MS method, we constructed calibration curves using data obtained from repeated injections of sets of standards in 12 amounts ranging from 1.0 fmol to 500 pmol. We also applied the stable isotope dilution method, comparing response ratios for each pair of unlabelled and labelled compounds. The responses covered a very broad linear range, spanning at least four orders of magnitude with correlation coefficients (*R*^2^) exceeding 0.9975 (Supplementary Table S2), in accordance with previously published linear ranges for LC-MS/MS methods (Matsuda *et al.*, 2005; Pěnčík *et al.*, 2009; Floková *et al.*, 2014). In the optimized MRM mode, the LODs ranged from 2.5 fmol to 50 fmol (Supplementary Table S2). Overall, the method’s sensitivity allowed the straightforward analysis and determination of IAA metabolites in extracts of just 2.0 mg FW of Arabidopsis tissue, and >250 samples could be quantified per day.

### Validation of the profiling method

The effectiveness of the method was validated by spiking experiments. The results confirmed the high precision and accuracy of the method (Supplementary Fig. S2B). The mean precision obtained in the spiking experiments with Arabidopsis extracts was 6 ± 3% RSD and the mean accuracy for all compounds was 1 ± 17% BIAS (percentage deviation from the accepted reference value), confirming the robustness of our method.

We also processed an extract from 7-day-old Arabidopsis seedlings using both our new µSPE-based method and the previously published one-step purification protocol, involving purification on reversed-phase Oasis™ HLB columns (Novák *et al.*, 2012).

Levels of IAA and its metabolites (precursors, catabolites, and conjugates), analysed by ultra-fast LC-MS/MS and quantified by standard isotope dilution, following the two purification methods, were similar (Table 1). Furthermore, profiles and levels of most known auxin precursors and conjugates/catabolites were very similar to previously published patterns (Kai *et al.*, 2007; Novák *et al.*, 2012). The miniaturized SPE system also provided substantially better method precision (Table 1) than the commercially available polymeric HLB columns, with an overall mean of 3.4% RSD compared with 4.7% RSD. However, it was not possible to calculate IAOx and TRA levels in extracts purified by StageTips microcolumns, probably due to their very low endogenous levels, lower capacity of C_18_/SDB-XC sorbents (Fig. 2), and/or strong effects of the plant matrix.

**Table 1.** Levels of IAA metabolites in a 7-day-old Arabidopsis Col-0 extract (10 mg FW of tissue extracted in 1 ml of 50 mM Na-phosphate buffer, pH 7.0 and quantified by LC-MRM-MS after purification by in-tip µSPE or HLB columns)

Compound	Content of IAA metabolites (pmol g^−1^ FW)	
	In-tip µSPE	HLB
ANT	297 ± 27	318 ± 17
TRP	97751 ± 2833	99522 ± 7618
TRA	ND	13 ± 4
IAM	9 ± 1	8 ± 1
IAN	56792 ± 1053	74042 ± 1851
IAOx	ND	ND
IPyA	158 ± 11	133 ± 7
IAA	243 ± 2	243 ± 5
OxIAA	1717 ± 26	1611 ± 37
IAAsp	55 ± 5	48 ± 3
IAGlu	52 ± 1	49 ± 4
IAA-glc	360 ± 3	382 ± 19
OxIAA-glc	6918 ± 41	7039 ± 43

Values are means ±SD (*n*=4); ND, not detected.

Taken together, our results in these validation experiments demonstrate the accuracy and robustness of our high-throughput method for routine determination of key auxin precursors and conjugates/catabolites in minute samples of plant material (<10 mg FW).

### High-throughput mutant screening of Arabidopsis lines

Next, we tested the ability of our method to identify genotypes with abnormal IAA metabolite profiles within a collection of Arabidopsis mutant lines. We chose 64 lines that were initially isolated based on perturbations in their leaf morphology (Berná *et al.*, 1999; Pérez-Pérez *et al.*, 2009). The L*er* accession, which is the genetic background of the mutants, was used as a wild-type control for the analysis, and the auxin-overproducing mutant line *sur2-1* (Barlier *et al.*, 2000), which has an altered IAA metabolite profile compared with L*er* (Supplementary Fig. S4), was included among the mutant lines as a positive control.

High-throughput auxin metabolite profiling, combining the one-step in-tip µSPE purification protocol and ultra-fast LC-MS/MS analysis (Fig. 2C), was performed on a total of 365 samples (five biological replicates per mutant line and 40 biological replicates of the L*er* wild-type; see the Materials and methods). This approach allowed multiplex quantification of IAA and its precursors (ANT, Trp, IPyA, and IAN) and degradation products (oxIAA, IAAsp, IAGlu, IAA-glc, and oxIAA-glc), but not (under our method conditions) of TRA and IAM, as their signal intensities were consistently below their respective LODs, and of IAOx, as this metabolite was detected in only 10 out of 66 analysed genotypes.

**Fig. 3.**
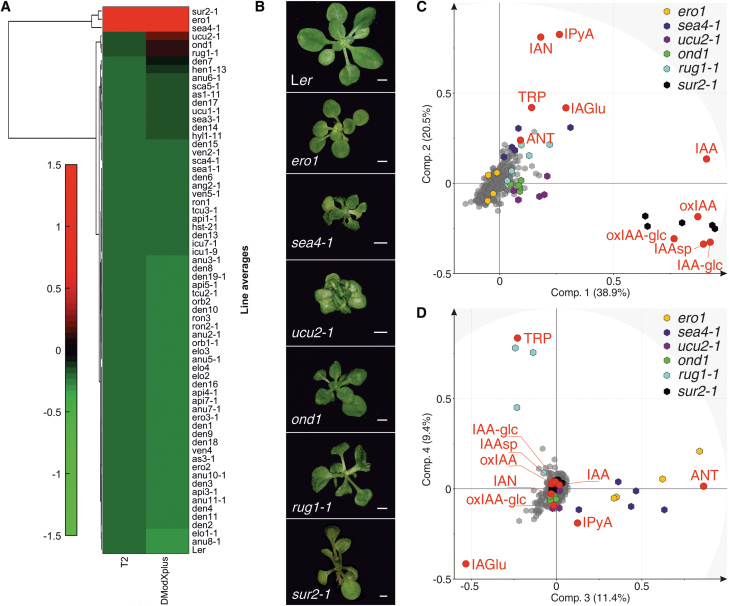
Identification of five mutant lines with markedly different IAA metabolic profiles. (A) Clustergram of average normalized Hotelling’s distance (T2) and distance to the model (DModXP+) values relative to the PCA model for the L*er* wild-type IAA metabolite profile (for which values for all variables are zero). Euclidean distance was used for lines, linear correlation for variables (metabolites), and average linkage for both. Green and red indicate the degree of similarity to and difference from L*er*, respectively. Values were calculated with SIMCA and the clustergram was constructed in MATLAB. (B) Rosette phenotypes of the L*er* wild type and the five most different lines identified in the analysis, 20 d after stratification (das). The *sur2-1* mutant was photographed on 16 das. Scale bars represent 1 mm. (C, D) PCA biplots showing separation of the samples (mutant lines, represented by hexagons) according to the variables (metabolites, represented by red dots). Points indicating lines with the highest degree of separation are coloured in yellow (*ero1*), dark blue (*sea4-1*), light blue (*rug1-1*), violet (*ucu2-1*), green (*ond1*), and black (*sur2-1* control line). Points indicating the other lines, including the L*er* wild type, are coloured grey. Biplots were constructed in SIMCA and correspond to (B) PC1 versus PC2 and (C) PC3 versus PC4, together explaining 80% of the total variation in the data.

MVDA was then applied to the data set to identify mutant lines with different IAA metabolite profiles from the L*er* wild type. Clustering of the lines’ average concentrations of metabolites revealed high diversity in the profiles (Supplementary Fig. S5), as expected for mutants isolated based on a criterion (perturbations of leaf architecture) that might be associated with diverse metabolic phenotypes. At the same time, such variety in the profiles provides an indication of the high interconnection of the IAA metabolome with many different processes, which might be represented among the mutant lines. Nevertheless, our principal component analysis (PCA) revealed a few lines that are remarkably different from the wild type in terms of their IAA metabolite profiles (Fig. 3). Clustering of the Hotelling distance (T2) and distance to the model (DModXP+) values, which indicate how far a line is from the wild type in different PCA models, ranked the lines by their similarity to L*er*, and identified those which were most different (Fig. 3A, top lines). These lines (*ero1*, *sea4-1*, *ucu2-1*, *ond1*, and *rug1-1*; Fig. 3B) separated from other samples, mainly due to their different contents of TRP, ANT, IAN, IPyA, IAA, oxIAA, and oxIAA-glc (Fig. 3C, D; Supplementary Fig. S6). As expected, the *sur2-1* mutant (Fig. 3B) was also found to be very different from the wild type (Fig. 3A) and markedly separated from other lines due to differences particularly in IAA, IPyA, IAN, oxIAA, IAAsp, IAA-glc, and oxIAA-glc levels (Fig. 3C). This is consistent with previously published data from auxin metabolite profiling of the *sur2-1* mutant line by Novák *et al.* (2012), and corroborates the accuracy of our analytical method.

**Fig. 4.**
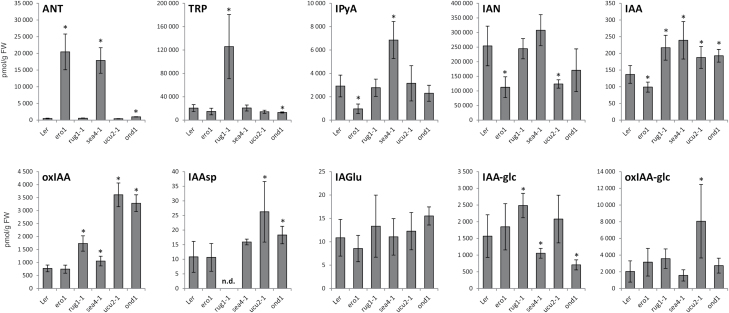
IAA metabolite concentrations in the L*er* wild type and the *ero1*, *rug1-1*, *sea4-1*, *ucu2-1*, and *ond1* mutants. Seedlings of wild-type Arabidopsis L*er* and mutant lines were collected in five replicates of 10 mg, and the IAA and IAA metabolites were analysed by LC-MS/MS. Error bars represent the SD. Asterisks indicate a statistically significant difference in a two-tailed Student *t*-test at a significance level of 0.01.

As shown in Fig. 4, metabolite profiles of the most different lines were very different from that of the L*er* wild type. The observed differences in IAA metabolite profiles also provide indications of the IAA metabolic pathways affected in these mutant lines (Fig. 1). However, an altered IAA metabolite profile can be a cause (e.g. when the function of an enzyme associated with IAA metabolism, or a regulator of such enzyme, is perturbed) or a consequence (e.g. the result of any other misfunction that indirectly changes the IAA metabolome). It is fair to assume that perturbations in direct regulators of IAA metabolism will create the greatest alterations in the IAA metabolite profile. The *sur2-1* mutant, which directly perturbs IAA metabolism, as it impairs the function of the cytochrome P450 monooxygenase CYP83B1 that regulates the levels of the IAA precursor IAOx (Barlier *et al.*, 2000; Bak *et al.*, 2001), was found to be the most different line in terms of its IAA metabolite profile (Fig. 3A–C). Together with *sur2-1*, the *ero1* and *sea4-1* mutants were most separated from the wild type and other lines (Fig. 3A–C), which raises the possibility that *ERO1* and *SEA4* genes are also involved in direct regulation of IAA metabolism.

However, only the mutated genes in the *rug1-1* and *ucu2-1* mutants have been identified (Pérez-Pérez *et al.*, 2004; Quesada *et al.*, 2013), not those in the *ero1*, *sea4-1*, and *ond1* mutants. The *RUG1* gene encodes porphobilinogen deaminase, an enzyme of the tetrapyrrole biosynthetic pathway. *rug1-1* mutant plants accumulate porphobilinogen, and both their vegetative and reproductive development is perturbed (Quesada *et al.*, 2013). We found that the *rug1-1* mutant also exhibited elevated levels of IAA, its precursor TRP, and the downstream products oxIAA and IAA-glc (Figs 1, 4). The particularly high levels of TRP in *rug1-1* plants suggests a link between the tetrapyrrole pathway, TRP biosynthesis, and IAA homeostasis.

The *ucu2-1* mutation, which causes increases in levels of IAA, IAAsp, oxIAA, and oxIAA-glc (Figs 1, 4), perturbs AtFKBP42, a peptidyl-prolyl *cis*-*trans* isomerase of the FK506-binding protein family that participates in auxin and brassinosteroid signalling (Pérez-Pérez *et al.*, 2004). AtFKBP42 also mediates polar auxin transport through its requirement for proper localization of the ABCB/PGP-efflux carriers to the plasma membrane (Wu *et al.*, 2010; Henrichs *et al.*, 2012). As found in this study, inefficient ABCB/PGP-mediated auxin efflux results in elevated levels of IAA and IAA degradation products in the *ucu2-1* plants (Fig. 4), probably caused by feedback promotion of IAA biosynthesis in the sink tissues, devoid of auxin as a result of the IAA gradient disruption in the mutant (Wu *et al.*, 2010). A detailed study of the lines identified by the present approach might reveal new players in the IAA metabolic pathways, as well as new interconnections between IAA metabolism and other pathways operating in plants.

### Phenotypical analysis of the mutant lines

**Fig. 5.**
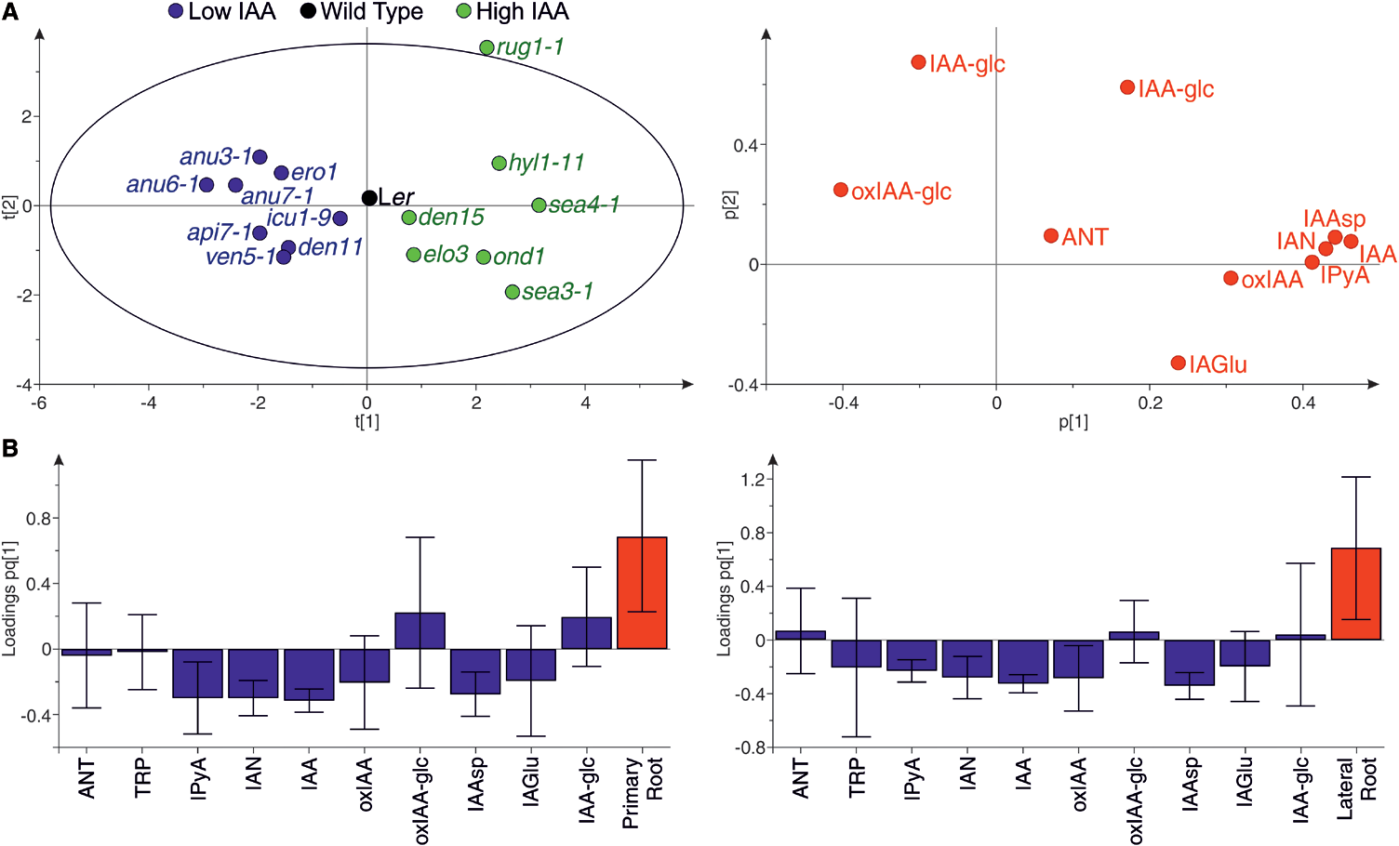
Relationship between IAA metabolites and auxin-related phenotypes in selected lines. (A) PCA on the selected lines in Supplementary Fig. S7 and 10 IAA metabolites. The scatter plot of the scores (left) shows the separation of the lines with higher (green) and lower (blue) IAA levels than in the wild type, based on the metabolites shown in the scatter plot of the loadings (right). The percentage of the variance explained by the components t1 and t2 is 41.9% and 16.5%, respectively. (B) OPLS predictive loadings for PCA models of the primary root length (left) and lateral root density (right) using the selected lines in Supplementary Fig. S7 and 10 IAA metabolites. Model statistics for primary root: one latent variable; R2X=0.377; R2Y=0.292; Q2=0.129. Model statistics for lateral roots: one latent variable; R2X=0.368; R2Y=0.307; Q2=0.049.

To explore the relationship between IAA and the levels of other IAA metabolites among the lines, we focused on the endogenous IAA contents of the 64 mutant lines, and selected eight lines with lower and seven lines with higher IAA contents than the wild type (Supplementary Fig. S7A). PCA on the selected lines with low and high IAA levels revealed that, together with IAA, a major component explaining the separation of the lines is IAAsp, IAN, and IPyA on one side, and IAA-glc and oxIAA-glc on the other side (Fig. 5A). This indicates, regardless of the phenotype of the mutants, a behaviour of the IAA metablome in which IAAsp, IAN, and IPyA levels are positively correlated, while IAA-glc and oxIAA-glc levels are negatively correlated with IAA levels in these particular lines (Fig. 5A).

We then explored how the IAA metabolite profiles correlated with IAA-related phenotypes. We determined the length of the primary root and the hypocotyl, as well as the density of lateral roots, in the selected lines (Supplementary Fig. S7B–D), as these phenotypes are highly dependent on IAA levels (Porco *et al.*, 2016; Zheng *et al.*, 2016). Orthogonal Projections to Latent Structures (OPLS) models were constructed using the phenotypic measurements and the metabolite concentrations in these lines. While no model could be generated for the hypocotyl length, due to the lack of correlation of this phenotype with the IAA metabolite profiles in these specific lines, OPLS models for primary root length and lateral root density were generated (Fig. 5B). These two models show very similar patterns, in which IAA, together with IPyA, IAN, oxIAA, and IAAsp, levels are negatively correlated with the primary root length and the lateral root density in the lines examined (Fig. 5B). The fact that these metabolites are negatively correlated with these phenotypes should be understood, however, as a reflection of the positive correlation between IAA and IPyA, IAN, oxIAA, and IAAsp (Fig. 5A), which is the bioactive metabolite. Finally, it is important to note here that OPLS models were constructed based on metabolite profiles from whole Arabidopsis seedlings, whilst morphological phenotypes may depend on local increases or decreases in IAA levels. Tissue-specific IAA metabolite profiling should be the choice to create stronger models from which to draw solid conclusions about the relationship between the IAA metabolome and specific developmental phenotypes.

In summary, the method presented here is a powerful tool for rapid IAA metabolome screening, which provides a valuable resource of informative data and facilitates the identification of novel regulators of IAA metabolism. It is important to note, however, that the IAA metabolome can be significantly perturbed by many different processes, as suggested by the *rug1-1* and *ucu2-1* mutants and by the huge diversity of IAA profiles among the studied genotypes (Supplementary Fig. S5). A case-by-case study of selected mutants after PCA is decisive to discern between direct and indirect effects.

### Concluding remarks

Overall, the results show a high potential of our method for powerful, rapid metabolome-based screening to measure IAA metabolites, characterize the pathways and the interactions involved, and to help identify novel regulators of the homeostasis of IAA and its metabolites. We have developed a robust ultra-rapid MS-based approach for extracting, purifying, and quantifying most known IAA metabolites, including IAA and its key precursors and conjugates/catabolites, from minute plant samples for high-throughput mutant screening. Sample extraction and micro-scale purification conditions were optimized using a selection of appropriate sorbent types. The method was tested using a collection of previously isolated mutant lines. Affording analysis of >100 samples per day, the procedure is less time-consuming and much more effective than previously published methods. It will allow researchers to quantify auxin metabolites, in large numbers of samples containing a few milligrams of fresh plant material, highly accurately and reproducibly. In addition, the reduction in the amount of plant material greatly facilitates a tissue-specific metabolome-based screening of IAA and IAA metabolites. In combination with multivariate data analysis, the method provides a powerful tool for mutant screening and potential identification of novel regulators of the IAA metabolism.

Furthermore, the same approach could be applied to other classes of plant hormones and metabolites, and used for high-throughput metabolic phenotyping of plants with different genetic backgrounds (e.g. knockout and overexpressing mutant and transgenic lines). The application of the present method to wild-type accessions or crop cultivars will be of great use for gene discovery (e.g. in genome-wide association studies based on hormone metabolomes) and for breeding programmes. Finally, we highlight the importance of interdisciplinary collaboration between plant biologists, mathematicians, and chemists for advancing our understanding of IAA and its functions as a crucial plant growth regulator.

## Supplementary data

Supplementary data are available at *JXB* online.

Fig. S1. Effects of tested loading conditions on the recovery (%) of IAA metabolites after purification by in-tip μSPE.

Fig. S2. Method optimization and validation.

Fig. S3. Comparison of signal sensitivities of IAA-glc and oxIAA-glc in analyses by LC-MS/MS using negative-ion (ESI–) and positive-ion (ESI+) multireaction monitoring (MRM) modes.

Fig. S4. IAA metabolite profiles in 7-day-old Arabidopsis seedlings of the wild-type accessions Landsberg *erecta* (L*er*) and Columbia (Col-0), and the IAA-overproducing mutant line *sur2-1*.

Fig. S5. High-throughput IAA metabolite profiling of the lines.

Fig. S6. Separation of the mutant lines according to their IAA metabolite levels.

Fig. S7. Auxin-related phenotypes in the Arabidopsis mutant lines selected by an ultra-rapid auxin metabolite profiling method.

Table S1. Stability of IAA-glc and oxIAA-glc in indicated solutions with pH 3–12, 0.1% acetic acid and 80% methanol.

Table S2. Diagnostic MRM transitions, optimized collision energies, retention time stability, limits of detection (LOD), dynamic linear range, and linearity (correlation coefficients, *R*^2^) of the LC-MRM-MS method.

Supplementary Tables S1-S2+Figures S1-S7

## Author contributions

AP, RC, KL, and ON conceived the original idea; AP, RC, and VP carried out the experiments; AŽ synthesized IAA standards; RP performed multivariate data analysis; JLM provided a collection of Arabidopsis mutant lines; AP and RC wrote the manuscript with support from JLM, KL, and ON; and KL and ON planned the experiments and supervised the project. All authors discussed the results and contributed to the final manuscript.
