# Supplementary material for: Ultra-rapid auxin metabolite profiling for high-throughput mutant screening in Arabidopsis
Source: J Exp Bot. 2018 Mar 3;69(10):2569–79. doi: 10.1093/jxb/ery084 (PMC5920284; doi:10.1093/jxb/ery084)
Supplement: Supplementary Tables S1-S2+Figures S1-S7 [file ery084_suppl_supplementary_tables_s1-s2+figures_s1-s7.pdf]

Supplementary data

Ultra-rapid auxin metabolite profiling for high-throughput Arabidopsis mutant screening

Aleš Pěnčík, Rubén Casanova Sáez, Veronika Pilařová, Asta Žukauskaitė, Rui Pinto, José Luis Micol, Karin Ljung and Ondřej Novák

Table S1. Stability of IAA-glc and oxIAA-glc in indicated solutions with pH 3 – 12, 0.1% acetic acid and 80% methanol.

Compound	pH < 3.0	pH = 4.0	pH = 8.0	pH > 12.0	0.1% acetic acid	80% MeOH
IAA-glc	+++	+++	+	–	+++	+++
oxIAA-glc	+++	+++	+	–	+++	+++

Recovery: “–“, 0-10%; “+”, 10-50%; “++”, 50-90%; “+++”, 90-100%; Mean ± SD (n = 4).

Table S2. Diagnostic MRM transitions, optimized collision energies, retention time stability, limits of detection (LOD), dynamic linear range and linearity (correlation coefficients, R^2) of the LC-MRM-MS method.

Optimized MS conditions were as follows: Drying Gas Temperature, 150°C; Drying Gas Flow, 16 l min^{-1}; Nebulizer Pressure, 40 psi; Sheath Gas Temperature, 375°C, Sheath Gas Flow, 12 l min^{-1}; Capillary Voltage, 3400 V; Nozzle Voltage, 0 V; Delta iFunnel High/Low Pressure RF, 110/60 V; and Fragmentor, 380 V.

Compound	MRM transition	IS[a]	MRM transition	CE[b] (V)	Retention time[c] (min)	LOD[d] (fmol)	Linear range (pmol)	R^2
Trp	205.2 > 146.1	[$^2H_5$]-Trp	210.2 > 151.1	14	0.931 ± 0.004	10.0	0.050-500	0.9987
TRA	161.1 > 144.1	[$^2H_2$]-TRA	163.1 > 146.1	6	0.932 ± 0.008	10.0	0.050-50	0.9998
IPyA-TAZ	263.1 > 88.0	[$^2H_4$]-IPyA-TAZ	267.1 > 88.0	10	1.617 ± 0.006	5.0	0.010-100	0.9981
ANT	138.1 > 120.1	[$^{13}C_6$]-ANT	144.1 > 126.1	6	1.728 ± 0.004	10.0	0.050-500	0.9999
oxIAA-glc	192.1 > 146.1	[$^{13}C_6$]-oxIAA-glc	198.1 > 152.1	12	1.759 ± 0.005	50.0	0.100-100	0.9991
IAM	175.1 > 130.1	[$^2H_5$]-IAM	180.1 > 135.1	12	1.842 ± 0.003	2.5	0.005-500	0.9999
IAAsp	291.2 > 130.1	[$^{13}C_6$]-IAAsp	297.2 > 136.1	20	2.027 ± 0.016	25.0	0.050-500	0.9999
oxIAA	192.1 > 146.1	[$^{13}C_6$]-oxIAA	198.1 > 152.1	12	2.064 ± 0.002	5.0	0.050-500	0.9996
IAA-glc	176.1 > 130.1	[$^{13}C_6$]-IAA-glc	182.1 > 136.1	12	2.165 ± 0.003	50.0	0.100-100	0.9976
IAGlu	305.2 > 130.1	[$^{13}C_6$]-IAGlu	311.2 > 136.1	20	2.294 ± 0.009	5.0	0.050-500	0.9997
IAA	176.1 > 130.1	[$^{13}C_6$]-IAA	182.1 > 136.1	12	2.822 ± 0.002	5.0	0.005-500	0.9999
trans-IAOx	175.1 > 158.1	[$^2H_5$]-*t*IAOx	180.1 > 163.1	10	3.011 ± 0.004	5.0	0.005-500	0.9978
IAN	157.1 > 130.1	[$^{13}C_6$]-IAN	163.1 > 136.1	10	3.185 ± 0.005	10.0	0.050-500	0.9991
cis-IAOx	175.1 > 158.1	[$^2H_5$]-*c*IAOx	180.1 > 163.1	10	3.258 ± 0.004	5.0	0.005-500	0.9982

[a] Internal standards used for stable isotope dilution.

[b] Collision energy.

[c] Mean ± SD (n=5).

[d] Signal-to-noise ratio of 3:1.

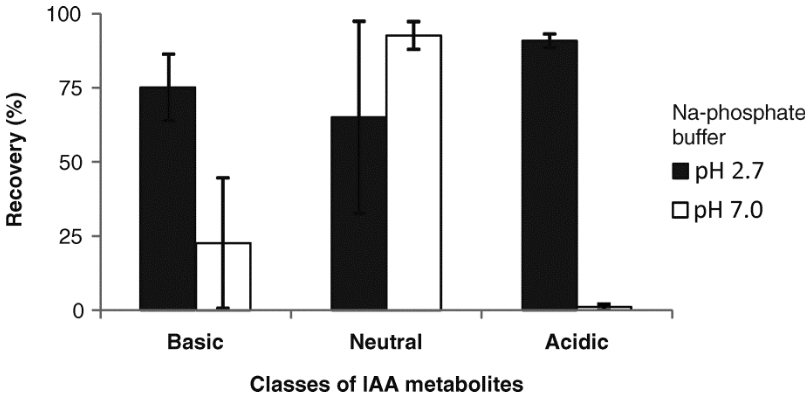

Figure S1. Effects of tested loading conditions on the recovery (%) of IAA metabolites after purification by in-tip μSPE.

The loading capacities were calculated as recoveries of the initial amount of each of the metabolites (1 pmol). Samples were analysed in four replicates. Error bars indicate standard deviations of the means (SD).

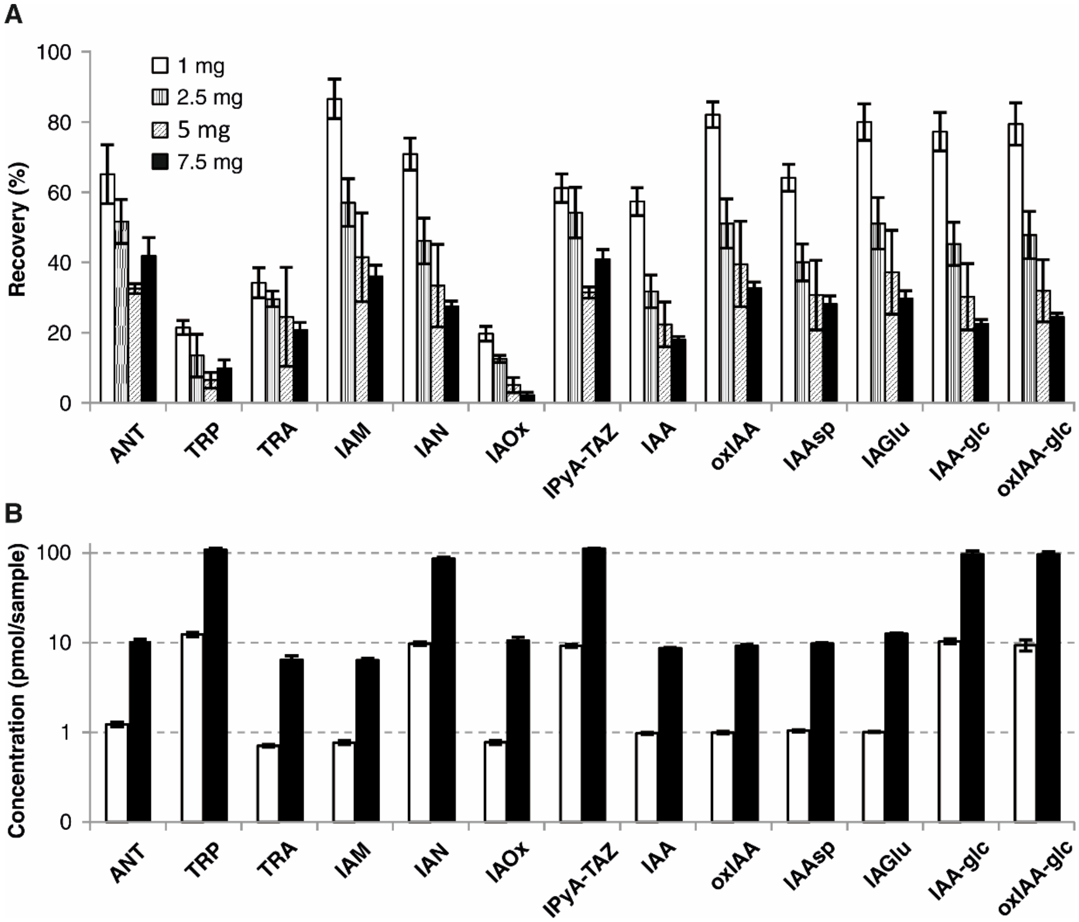

Figure S2. Method optimization and validation.

(A) Recoveries (%) of internal standards added to indicated amounts of Arabidopsis plant matrix (1 - 10 mg fresh weight) purified by multi-StageTips microcolumns packed with three layers of each sorbent (C18/SDB-XC). (B) Recovered amounts of IAA metabolites spiked in an Arabidopsis extract. Homogenous extract was divided into aliquots, each containing 2 mg of fresh tissue, supplemented with 1 and 10 pmol (white bars) or 10 and 100 pmol (black bars) of authentic standards (depending on the natural content of the respective metabolites) and a mixture of stable labelled internal standards (2.5 pmol of each, except for 5 pmol [$^{13}C_6$]-IAN and [$^2H_4$]-IPyA, and 50 pmol [$^2H_5$]-Trp). Samples were then purified by the one-step multi-μSPE protocol and subsequently analysed by LC-MS/MS. After subtracting endogenous auxin metabolite contents, concentrations of individual added metabolites were calculated. Samples were analysed in four replicates. Error bars indicate standard deviations of the means (SD).

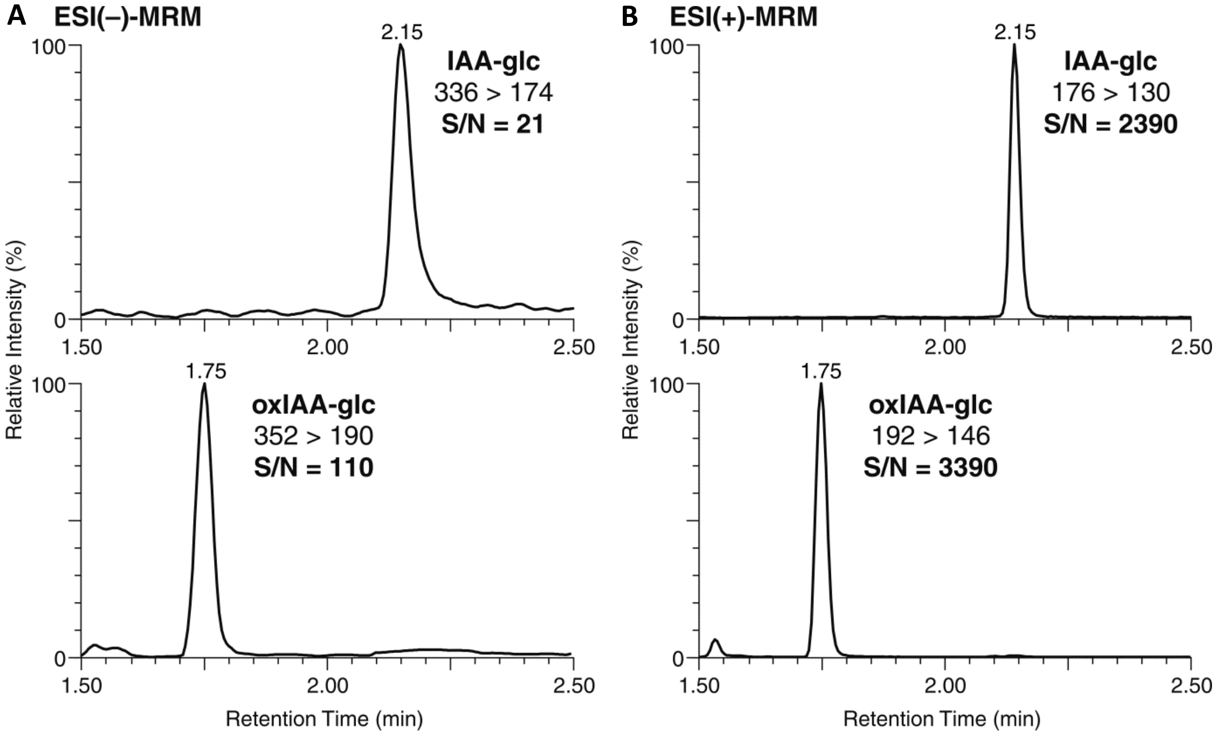

Figure S3. Comparison of signal sensitivities of IAA-glc and oxIAA-glc in analyses by LC-MS/MS using negative-ion (ESI–) and positive-ion (ESI+) multi reaction monitoring (MRM) modes.

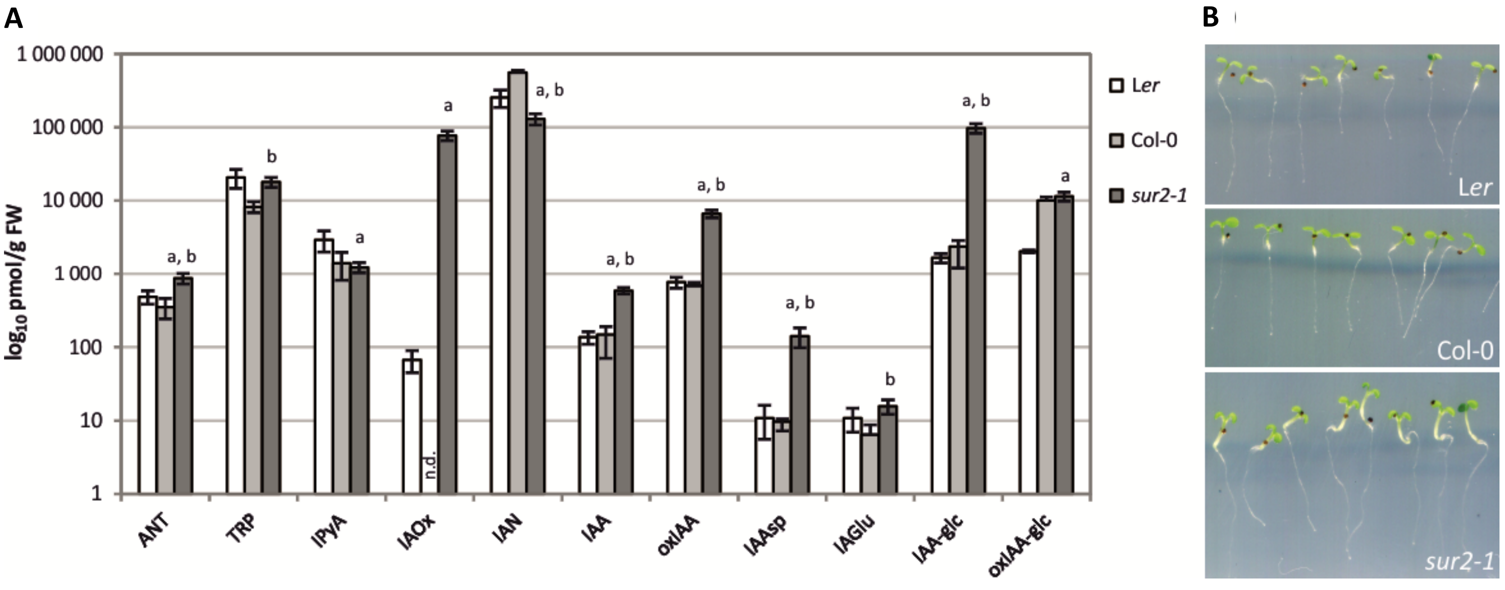

Figure S4. IAA metabolite profiles in 7-day-old Arabidopsis seedlings of the wild-type accessions Landsberg *erecta* (L*er*) and Columbia (Col-0) and the IAA over-producing mutant line *sur2-1*.

(A) Concentrations of all metabolites are plotted on a logarithmic scale. Error bars indicate SD (n = 5). Letters above the bars indicate statistically significant differences between (a) L*er* and *sur2-1*, and (b) Col-0 and *sur2-1* (two-tailed Student *t* test, P < 0.01). (B) Pictures to the right show corresponding 7-day-old seedlings of wild-type accessions (L*er* and Col-0) and the IAA over-producing mutant line *sur2-1*.

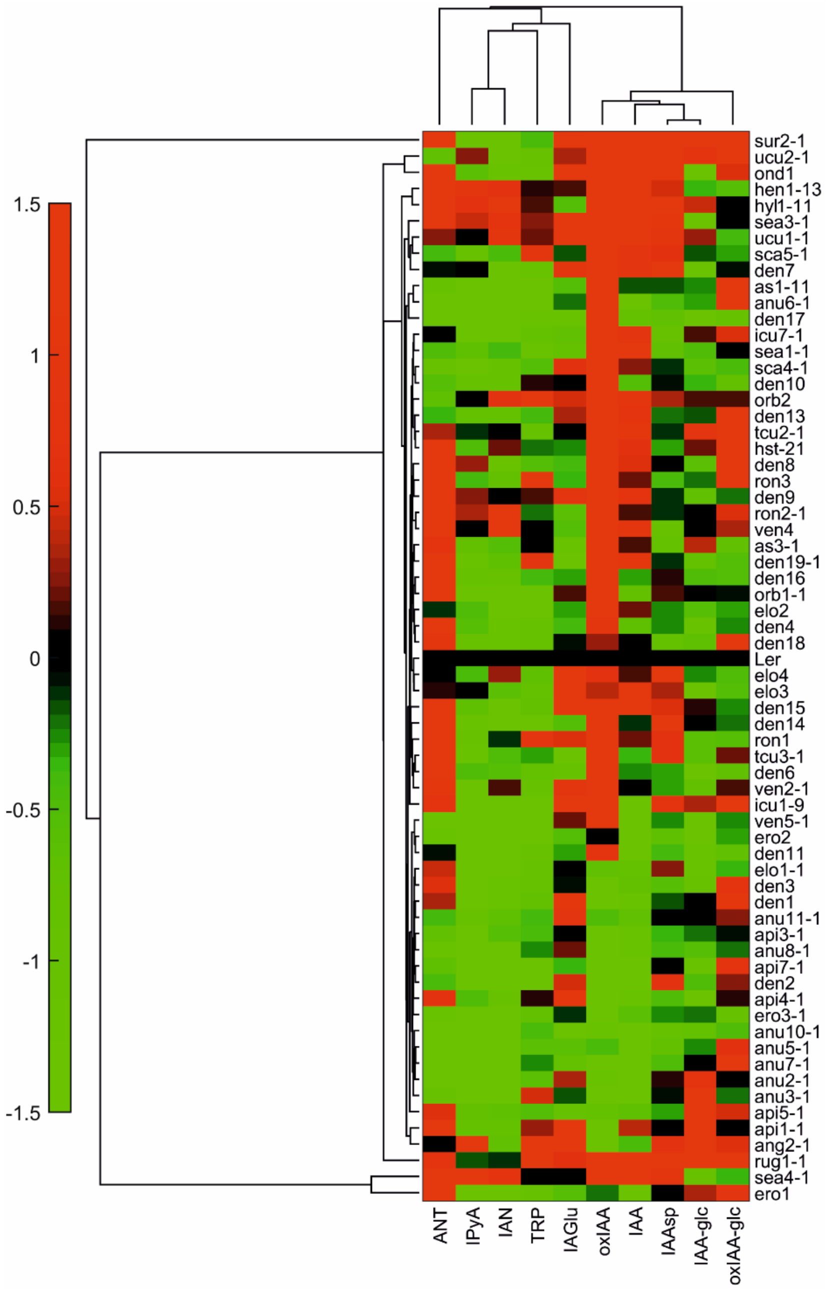

Figure S5. High-throughput IAA metabolite profiling of of the Arabidopsis mutant lines. Clustergram of the average concentration of the different metabolites in the 65 lines analyzed. Values are UV-scaled using the L*er* samples, so that the value of all variables for L*er* is zero. Euclidean distance was used to cluster lines, linear correlation for metabolites, and average linkage for both. Green and red colors indicate lower and higher concentrations compared to L*er*, respectively.

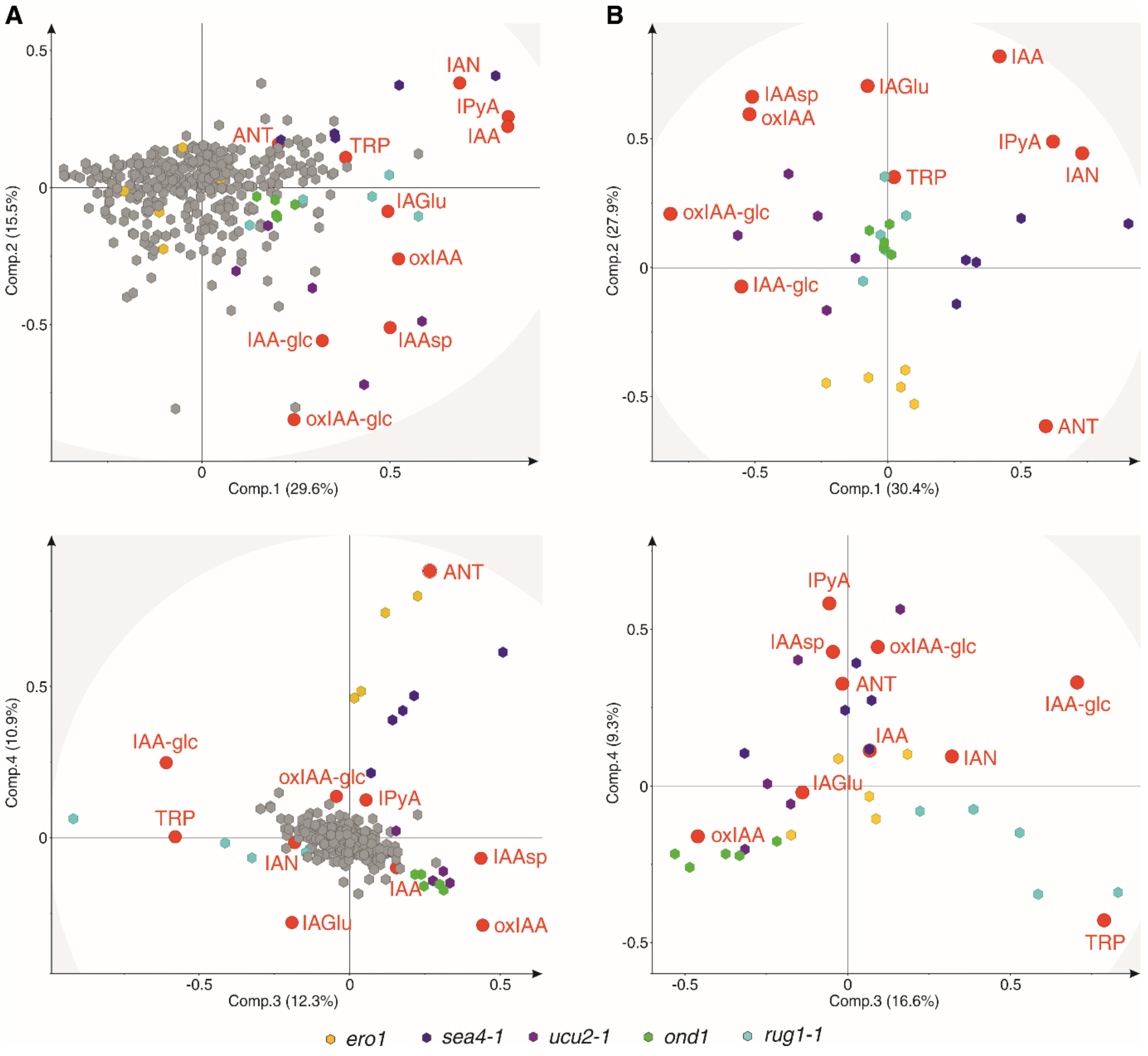

Figure S6. Separation of the mutant lines according to their IAA metabolite levels.

(A) PCA biplots showing the distribution of the mutant lines, excluding the *sur2-1* control line, based on components 1 vs.2 (top) and components 3 vs. 4 (bottom), together explaining 68.3% of the total variation of the data. Measured metabolites are indicated by red dots and mutant lines by hexagons. Lines that separated the most are coloured as indicated in the color panel; the others, including the L*er* wild type, are coloured in grey. (B) PCA biplots showing the distribution of only the most different lines shown in (a), based on components 1 vs. 2 (top) and components 3 vs. 4 (bottom), together explaining 84.2% of the total variation of the data. Measured metabolites are indicated by red dots and mutant lines by hexagons coloured as indicated in the color panel.

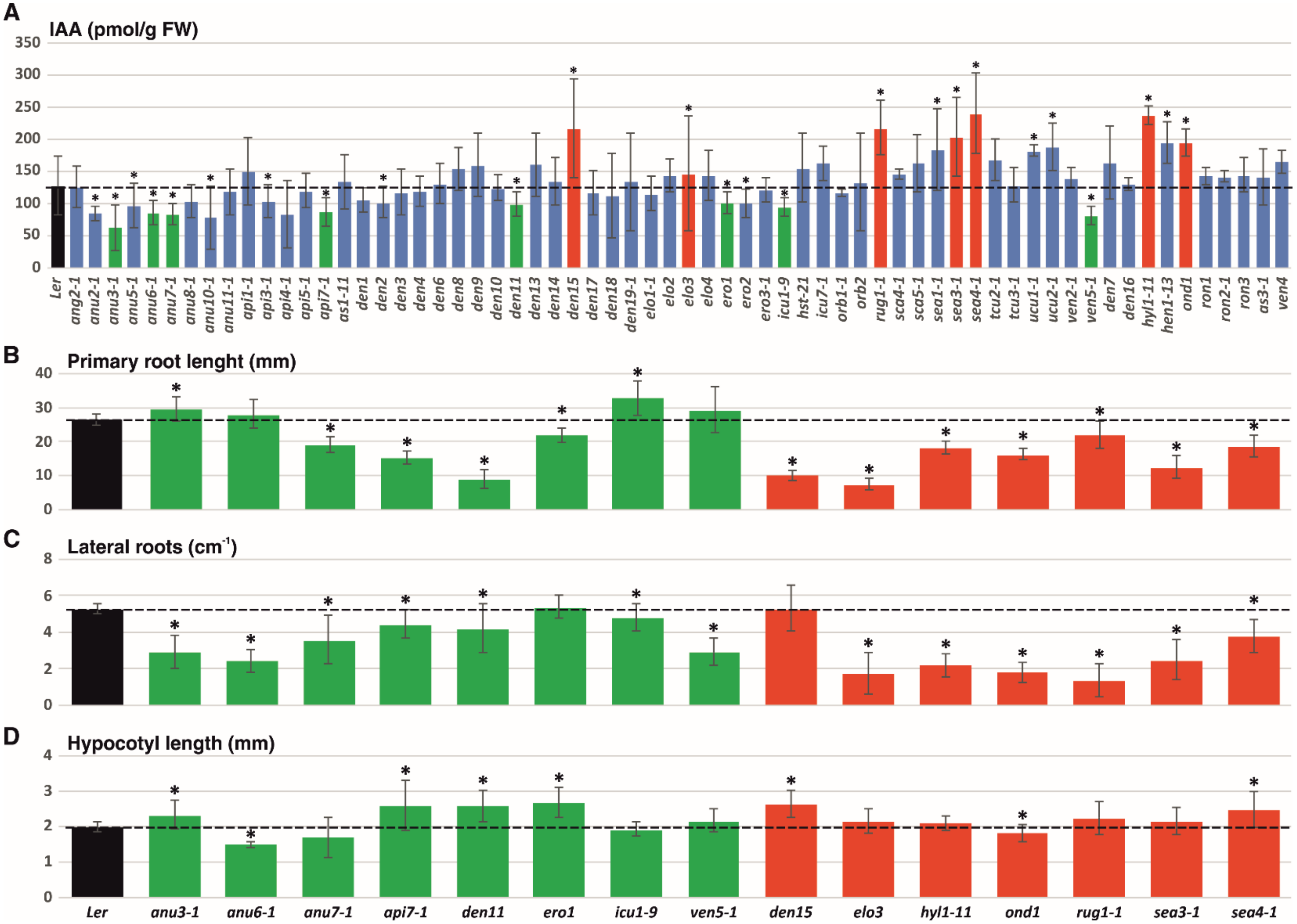

Figure S7. Auxin related phenotypes in the Arabidopsis mutant lines selected by an ultra-rapid auxin metabolite profiling method. (A) Concentration of IAA in 7-day-old seedlings from Ler and the 64 leaf mutants analysed in this study. Seedlings were collected in 5 replicates of 10 mg, and the IAA levels were analysed by LC-MS/MS. Error bars represent SD. Asterisks indicate statistically significant difference in a two-tailed Student t test at a significant level of 0.01. Selected lines with with lower (green) and higher (red) IAA levels compared to Ler are indicated. (B-C) Plant phenotyping. Length of the primary root (B), density of lateral roots (C) and length of the hypocotyl (C) in10-day-old seedlings from the selected lines in (A). Error bars represent SD (n = 10-14). Asterisks indicate statistically significant difference in a two-tailed Student t test at a significant level of 0.05. The average of the wild-type value is indicated with a black dashed line in all charts.
